# Isoprenoid-chained lipid EROCOC_17+4_: a new matrix for membrane protein crystallization and a crystal delivery medium in serial femtosecond crystallography

**DOI:** 10.1038/s41598-020-76277-x

**Published:** 2020-11-09

**Authors:** Kentaro Ihara, Masakatsu Hato, Takanori Nakane, Keitaro Yamashita, Tomomi Kimura-Someya, Toshiaki Hosaka, Yoshiko Ishizuka-Katsura, Rie Tanaka, Tomoyuki Tanaka, Michihiro Sugahara, Kunio Hirata, Masaki Yamamoto, Osamu Nureki, Kensuke Tono, Eriko Nango, So Iwata, Mikako Shirouzu

**Affiliations:** 1 RIKEN Center for Biosystems Dynamics Research, 1-7-22 Suehiro-cho, Tsurumi-ku, Yokohama, Kanagawa 230-0045 Japan; 2 Department of Biological Sciences, Graduate School of Science, The University of Tokyo, 7-3-1 Hongo, Bunkyo-ku, Tokyo, Japan; 3 RIKEN SPring-8 Center, 1-1-1, Kouto, Sayo-cho, Sayo-gun, Hyogo, 679-5148 Japan; 4 Department of Cell Biology, Graduate School of Medicine, Kyoto University, Yoshidakonoe-cho, Sakyo-ku, Kyoto, 606-8501 Japan; 5 Japan Synchrotron Radiation Research Institute, 1-1-1, Kouto, Sayo-cho, Sayo-gun, Hyogo, 679-5198 Japan; 6 Present Address: MRC Laboratory of Molecular Biology, Cambridge Biomedical Campus, Francis Crick Ave, Cambridge, CB2 0QH UK

**Keywords:** Structural biology, X-ray crystallography

## Abstract

In meso crystallization of membrane proteins relies on the use of lipids capable of forming a lipidic cubic phase (LCP). However, almost all previous crystallization trials have used monoacylglycerols, with 1-(*cis*-9-octadecanoyl)-*rac*-glycerol (MO) being the most widely used lipid. We now report that EROCOC_17+4_ mixed with 10% (w/w) cholesterol (Fig. 1) serves as a new matrix for crystallization and a crystal delivery medium in the serial femtosecond crystallography of Adenosine A_2A_ receptor (A_2A_R). The structures of EROCOC_17+4_-matrix grown A_2A_R crystals were determined at 2.0 Å resolution by serial synchrotron rotation crystallography at a cryogenic temperature, and at 1.8 Å by LCP-serial femtosecond crystallography, using an X-ray free-electron laser at 4 and 20 °C sample temperatures, and are comparable to the structure of the MO-matrix grown A_2A_R crystal (PDB ID: 4EIY). Moreover, X-ray scattering measurements indicated that the EROCOC_17+4_/water system did not form the crystalline L_C_ phase at least down to − 20 °C, in marked contrast to the equilibrium MO/water system, which transforms into the crystalline L_C_ phase below about 17 °C. As the L_C_ phase formation within the LCP-matrix causes difficulties in protein crystallography experiments in meso, this feature of EROCOC_17+4_ will expand the utility of the in meso method.

## Introduction

In meso crystallization of membrane proteins (MPs), a powerful technique for MP structure determination, critically relies on the choice of the lipids that form the LCP around room temperature^1,2^. In the method, a solubilized target MP is homogenized with an LCP-forming lipid, to uniformly reconstitute the MP into the native biomembrane mimetic LCP lipid bilayer. The added crystallization solution triggers the nucleation and crystal growth of the MP within the lipid bilayer environment, where the crystallization process essentially relies on the three-dimensional bi-continuous LCP bilayer architecture^2^. Moreover, the recent development of viscous media injectors^3^ has facilitated the direct use of a native crystal growth LCP-matrix as a crystal delivery medium in serial femtosecond or millisecond crystallography (SFX or SMX), known as LCP-SFX and LCP-SMX, respectively^4,5^.

**Figure 1:**
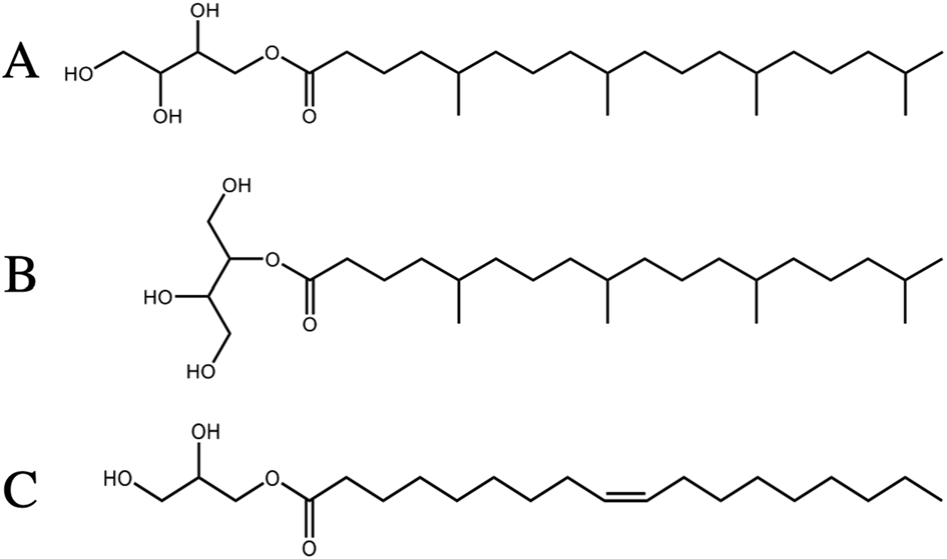
Chemical structure of 1-*O*-(5, 9, 13, 17-tetramethyloctadecanoyl)-*rac*-erythritol (**A**) and 2-*O*-(5, 9, 13, 17-tetramethyloctadecanoyl)-*rac*-erythritol (**B**). The lipid used in this study was a mixture of 92% 1-*O*- and 8% 2-*O*-isomers, which is abbreviated to EROCOC_17+4_. In the A_2A_R crystallization trials, a 9:1 (w/w) mixture of EROCOC_17+4_ and cholesterol was employed as a crystallization matrix, which is referred to as an EROCOC_17+4_-matrix (see Methods Crystallization section for more details). The isoprenoid chain is abbreviated as C_p+q_, where p and q stand for the number of carbon atoms in the longest unbranched carbon chain in the molecule (excluding the carbonyl carbon) and the number of methyl branches along it, respectively. Thus, EROCOC_17+4_ represents a lipid molecule, in which the C_17+4_ chain is linked to the erythritol head group (ER) via an ester linkage (OCO). (**C**) Chemical structure of 1-(*cis*-9-octadecanoyl)-*rac*-glycerol (MO). It is noted that EROCOC_17+4_ and MO both possess the acyl chain of 18 carbon atoms-long.

In spite of the increasing functional roles of the LCP, the currently available lipid species have been restricted by the limited choice of polar lipids. In fact, almost all of the past crystallization trials and LCP-SFX data collections have been performed by using monoacylglycerols (MAGs) at room temperature, with MO being the most widely used lipid^6^. However, MO is not ideal. For instance, according to the equilibrium phase diagram of the MO/water system, the MO-LCP gives way to the L_C_ phase below about 17 °C^7^. As LCP is prone to supercooling^8^, the MO-LCP may remain supercooled below 17 °C. However, as the MO-LCP is metastable below 17 °C, the risk always remains that the LCP → L_C_ phase transition could occur at any time during the experiment. The LCP → L_C_ phase transition incurs numerous difficulties. For example, the L_C_ phase does not support MP crystallization, and in LCP-SFX, when microcrystals randomly dispersed in the MO-LCP are injected into an evacuated X-ray diffraction chamber at 20 °C, evaporative cooling causes transformation into the MO-L_C_ phase. This leads to strong MO-L_C_ Bragg diffractions from crystallized hydrocarbon chains and three-dimensional orders in the L_C_ molecular packing, which interfere with the diffraction spots from MP crystals^3^. As background reduction is crucial in serial microcrystallography^9^, there is a need to develop new lipid matrices that support MP crystallization and do not undergo the LCP → L_C_ phase transition in LCP-SFX^4^. We now report that one of the LCP-forming isoprenoid-chained lipids (IPCLs), EROCOC_17+4_ with the acyl chain of 18 carbon atoms-long^10,11^ (see Fig. 1 for more details), serves as a new matrix lipid that satisfies the above requirements.

The recently developed LCP-forming IPCLs^10–14^, are characterized by their regularly methyl-branched chain structure (oligomers of a **–**(CH_2_)_2_CHCH_3_CH_2_**–** unit with a terminal **–**(CH_2_)_2_CHCH_3_CH_3_ unit, see Fig. 1), which is remarkably different from that of MAGs, consisting of different lengths of linear hydrocarbon chains with a *cis*-double bond at various chain positions (see Fig. 1)^6^. Owing to the regularly branched chain structure, IPCL-LCPs are characterized by the low L_C_ → LCP phase transition temperature (*T*_*K*_): most of the *T*_*K*_ values are close to or below 0 °C, as shown in Supplementary Table 1^10–14^. Thus, they are attractive as a new crystallization matrix lipid for temperature-sensitive proteins and as an L_C_ phase formation-free crystal delivery medium in LCP-SFX. However, to the best of our knowledge, only two structural analyses of bacteriorhodopsin (bR) with IPCL-matrices have been reported so far. One used an ether-type β-XylOC_16+4_^13^, which yielded crystals at 2 Å resolution with a reduced crystallographic twin ratio^15^, and the other used an amide-type GlyNCOC_15+4_, where the successful crystallization was performed at both 20 and 4 °C^12^. However, the utility of IPCLs as a possible crystal delivery medium in LCP-SFX has not been evaluated.

To assess EROCOC_17+4_ as a possible matrix lipid for MP crystallization and a crystal delivery medium in LCP-SFX, we employed the G protein-coupled receptor A_2A_R as a model MP, considering that MO-matrix, a 9:1 (w/w) mixture of MO and cholesterol, grown high-resolution crystal structures with ordered lipids have been reported^16–19^. We now demonstrate that EROCOC_17+4_-matrix, a 9:1 (w/w) mixture of EROCOC_17+4_ and cholesterol (see Methods Crystallization section for more details), supports the crystallization of A_2A_R at 20 °C, yielding a high-resolution crystal structure comparable to that of the MO-matrix grown crystals at 1.8 Å resolution (PDB ID: 4EIY, hereafter referred to as the 4EIY model), and that EROCOC_17+4_-matrix allows us to perform the LCP-SFX data collection with a sample kept at 4 °C, without the L_C_ phase formation.

## Results and discussion

### ***Crystallization of A***_***2A***_***R in the EROCOC***_***17***+***4***_***-matrix***

**Figure 2:**
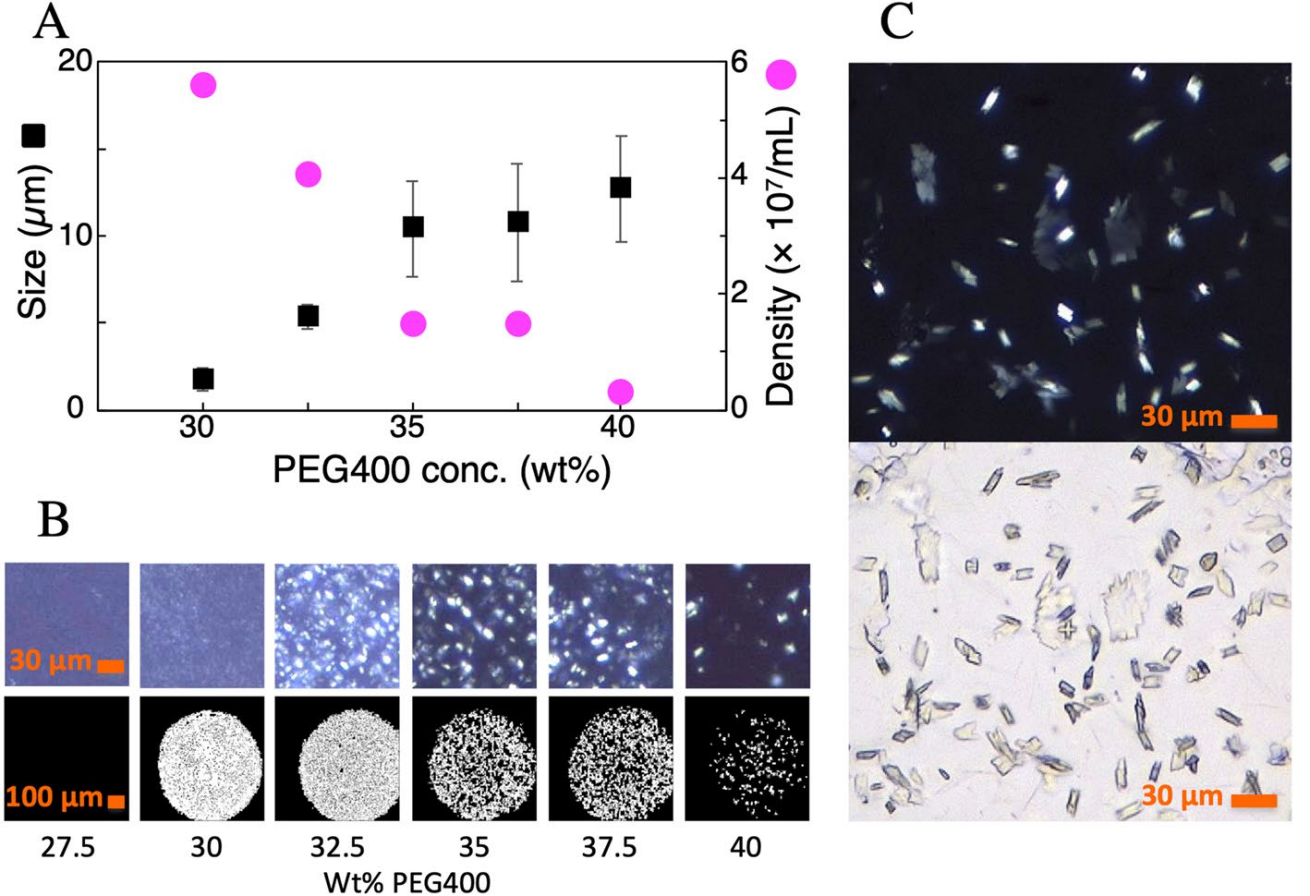
Effect of PEG concentration on size and density of A_2A_R crystals grown in the EROCOC_17+4_-matrix. (**A**) Size (black) and density (magenta) of EROCOC_17+4_-matrix grown A_2A_R crystals as a function of the PEG400 concentration, in an otherwise fixed solution composed of 50 mM NaSCN, 2% 2,5-hexanediol, and 100 mM Na citrate, pH 5.50. (**B**) Cross polarized (upper) and second harmonic generation (lower) images in a 96-well glass screening plate, showing the birefringence and chirality-derived signals of the protein crystals, respectively. (**C**) Crystals used for SS-ROX under optimized conditions: 38% PEG400, 50 mM NaSCN, 2% 2,5-hexanediol, and 100 mM Na citrate, pH 5.75. Upper: cross polarized, lower: bright field.

In the screening of crystallization conditions of A_2A_R as a model MP with the EROCOC_17+4_-matrix, we first noted that the optimal crystallization conditions for the MO-matrix, i.e., 100 mM sodium citrate, pH 5.0, 25–28% PEG400, 40–60 mM NaSCN, and 2% 2,5-hexanediol^16^, supported micro-crystal (≤ ~ 1 µm) formation in the EROCOC_17+4_-matrix at 20 °C. However, these crystals were too small for high-resolution structural analyses. To identify the optimum crystallizing conditions for the EROCOC_17+4_-matrix, we screened the effects of the pH (4.5–6.5 with 25–300 mM sodium citrate), and the concentrations of PEG400 or PEG300 (17.5–47.5%), NaCl (0–1000 mM), and the additives NaSCN (0–60 mM) and 2,5-hexanediol (0–2%), on the crystallization behavior, and noted that the most effective parameter was the PEG400 (or equivalently PEG300) concentration. The size and density of EROCOC_17+4_-matrix grown A_2A_R crystals as a function of the PEG400 concentration (*C*_*PEG400*_), in an otherwise fixed solution composition of 50 mM NaSCN, 2% 2,5-hexanediol, and 100 mM Na citrate, pH 5.50, are shown in Fig. 2A,B

The crystal size tended to increase as ${C}_{PEG400}$ increased; 3–10 µm at 30–35%, and eventually reaching ~ 15 μm sized-crystals at 35–40% PEG400, where the first crystals appeared at 1–2 days and continued to grow over ~ 1 week to reach the maximum size of ~ 25 μm (Fig. 2C), which is suitable for data collection by serial synchrotron rotation crystallography (SS-ROX)^20^.

On the other hand, the crystal density decreased as *C*_*PEG400*_ increased, from 10^7^–10^8^ crystals/mL around 30% down to 10^6^–10^7^ crystals/mL around 40%. The ${C}_{PEG400}$-dependent crystal size/density relation shown in Fig. 2A served as a useful guide for preparing ~ 50 μL of LCP densely packed with small crystals ($\ge {10}^{7}$ crystals/mL and 5–10 μm) needed for complete data collection by LCP-SFX. The final crystallization condition for the EROCOC_17+4_*-*matrix based LCP-SFX was 100 mM sodium citrate, pH 5.5, 37% PEG300, 50 mM NaSCN, and 2% 2,5-hexanediol. Thus, except for the PEG concentrations, the optimum crystallization conditions for A_2A_R in the EROCOC_17+4_-matrix did not differ significantly from those for the MO-matrix: the EROCOC_17+4_-matrix required 35–40% PEG400/PEG300, as compared to 25–28% PEG400 for the MO-matrix.

### Crystallographic analyses by SS-ROX and LCP-SFX

**Figure 3:**
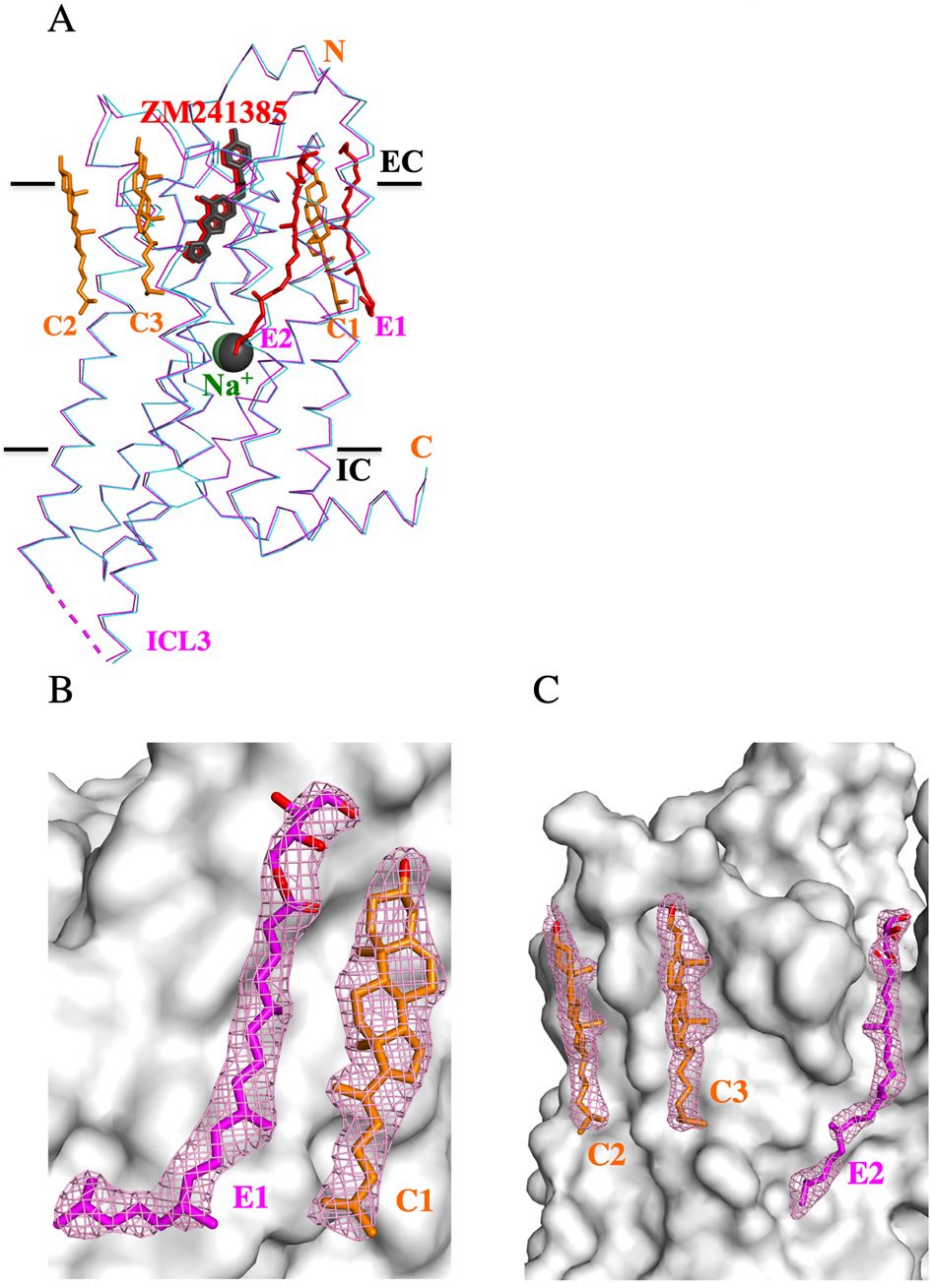
Comparison of EROCOC_17+4_- and MO-matrix grown A_2A_R crystal structures. (**A**) Crystal structures of LCP-SFX_4 °C (magenta) and 4EIY (cyan). The bRIL moiety inserted in ICL3 is omitted from the coordinates (magenta dashes). Sodium ions (green and dark grey balls for LCP-SFX_4 °C and 4EIY, respectively) and ZM241385 (red and dark grey sticks for LCP-SFX_4 °C and 4EIY, respectively) are also shown. Black lines approximately correspond to the hydrophobic/hydrophilic boundary of the lipid bilayer. IC and EC are intracellular and extracellular sides, respectively. Two well characterized EROCOC_17+4_ (red; E1 and E2) and three cholesterol (orange; C1, C2 and C3) models are displayed as sticks. (**B**,**C**) Molecular surface of LCP-SFX_4 °C with well characterized lipids. The 2m*F*_o_-D*F*_c_ electron density maps around the lipid models are contoured at 1.0 σ (pink mesh). The carbon atoms of the EROCOC_17+4_ and cholesterol stick models are shown in magenta and orange, respectively. The oxygen atoms of both lipids are shown in red. This figure is prepared by PyMOL Ver. 1.8 (https://pymol.org).

The crystallographic data of the EROCOC_17+4_-matrix grown A_2A_R crystals are summarized in Table 1. In the present SS-ROX and LCP-SFX experiments, we employed the A_2A_R crystals prepared at 20 °C. The samples for SS-ROX were flash frozen in SS-ROX_cryo, and those for the LCP-SFX loaded into the injector were kept at 4 or 20 °C during the measurements in LCP-SFX_4 °C and LCP-SFX_20 °C, respectively. The crystallographic space group of these EROCOC_17+4_-matrix grown crystals was the same as that for the MO-matrix grown crystals (PDB ID 4EIY), *C*222_1_. The structures of the EROCOC_17+4_-matrix grown A_2A_R crystals were determined at 2.0 Å resolution by SS-ROX at the cryogenic temperature, and at 1.8 Å by LCP-SFX_4 °C and LCP-SFX_20 °C, respectively. As in the cases described in the references^21,22^, no significant differences were observed in the electron density maps and the models obtained at the three temperatures, except for the increasing *B* factors and the decreasing number of water molecules at the higher temperatures (Table 1). The resolution of the LCP-SFX data was slightly better than that of the SS-ROX data for the present measurements, although the LCP-SFX and SS-ROX data are difficult to compare because the crystals and X-ray sources are different. On the other hand, comparing the LCP-SFX_4 °C and LCP-SFX_20 °C data obtained under exactly the same conditions, except for the sample temperatures, the LCP-SFX_4 °C model seems to be more reliable. The reliability factors, signal-to-noise ratio (SNR) values, and average *B* values for LCP-SFX_4 °C are slightly better than those for LCP-SFX_20 °C (Table 1). For example, the *R*_split/work/free_ values for LCP-SFX_4 °C are 6.6/17.5/20.7% whereas those of LCP-SFX_20 °C are 7.1/17.7/20.9%. Since the diffraction data could be improved by lowering the sample temperature, as described above, the LCP-SFX_4 °C model is used for further structure comparisons and analyses. The root mean square deviation (RMSD) value for the corresponding 299 Cα atoms between the LCP-SFX_4 °C and the MO-matrix based 4EIY model is 0.23 Å RMSD, and those for the inter Na^+^, a conserved sodium ion bound in A_2A_R, and ZM241385, a strong inverse agonist for A_2A_R, are 0.20 and 0.18 Å, respectively (Fig. 3A).

**Table 1:** Data collection and refinement statistics at three sample temperatures.

**Data**	LCP-SFX_20 °C	LCP-SFX_4 °C	SS-ROX_cryo
Sample temperatures (K)	293	277	100
PDB ID	6LPK	6LPJ	6LPL
**Data collection**			
Beamline	SACLA, BL3	SACLA, BL3	SPring-8, BL32XU
Wavelength (Å)	1.230	1.230	1.000
Crystal size (µm)	5–10	5–10	~ 15
Number of collected images	169,712	143,099	50,668
Number of hit images	34,216	65,791	6346
Number of indexed images	31,312	46,877	3701
Number of merged images	31,312	46,877	2100
Crystal hit rate (%)^a^	20.2	46.0	12.5
% indexed images (%)^b^	18.5	32.8	7.3
Crystal index rate (%)^c^	91.5	71.3	58.3
Cell constants a, b, c (Å)	40.5, 179.2, 142.9	40.4, 178.9, 142.4	39.9, 179.3, 141.0
Space group	C222_1_	C222_1_	C222_1_
Processed resolution (Å)	30.4–1.80 (1.83–1.80)	30.3–1.80 (1.83–1.80)	42.8–2.00 (2.08–2.00)
R_split_ (%)	7.1 (107.9)	6.6 (99.4)	9.0 (63.8)
CC_1/2_ (%)	99.5 (39.3)	99.6 (39.3)	99.1 (47.2)
Total measurements	14,994,871 (221,521)	16,847,719 (236,481)	1,729,208 (193,264)
Unique reflections	48,924 (2,438)	48,530 (2,390)	34,815 (3,765)
Completeness (%)	100 (100)	100 (100)	100 (100)
Multiplicity	306.5 (90.9)	344.7 (98.9)	49.7 (51.6)
SNR or averaged I/σ(I)	7.6 (1.0)	8.3 (1.1)	7.3 (1.9)
Wilson B value (Å^2^)	35.0	32.4	34.2
**Refinement**			
R_work_ (%)	17.7 (34.6)	17.5 (33.9)	17.3 (26.4)
R_free_ (%)	20.9 (35.8)	20.7 (35.7)	20.8 (24.2)
R.m.s. bond lengths (Å)	0.011	0.011	0.011
R.m.s. bond angles (°)	1.58	1.55	1.68
Overall B value (Å^2^):	56.2	51.8	49.0
B for A_2A_R-bRIL (Å^2^)	53.2	49.1	46.7
B for A_2A_R (Å^2^)	43.5	40.1	38.3
B for bRIL (Å^2^)	84.6	78.6	74.2
B for ZM241385 (Å^2^)	33.1	30.3	29.4
B for Na^+^ (Å^2^)	31.5	28.7	35.1
B for cholesterol (Å^2^)	55.5	50.2	48.0
B for EROCOC_17+4_ (Å^2^)	85.4	77.1	73.7
B for hydrocarbons (Å^2^)	85.7	79.1	71.9
B for water (Å^2^)	58.3	54.1	50.5
Number of amino acids in A_2A_R	299	299	299
Number of amino acids in bRIL	91	91	91
Number of ZM241385	1	1	1
Number of Na^+^	1	1	1
Number of cholesterol	3	3	3
Number of EROCOC_17+4_	2	2	2
Number of hydrocarbons	20	20	20
Number of water	116	123	138
Ramachandran favored	383 (99.2%)	384 (99.5%)	380 (98.4%)
Ramachandran allowed	3 (0.8%)	2 (0.5%)	6 (1.6%)
Ramachandran outlier	0 (0.0%)	0 (0.0%)	0 (0.0%)

^a^(Number of hit images/Number of collected images) × 100.

^b^(Number of indexed images/Number of collected images) × 100.

^c^(Number of indexed images/Number of hit images) × 100.

### ***Lipid molecules on the A***_***2A***_***R surface***

After the refinement of the A_2A_R structure, the electron density maps showed elongated extra electron densities surrounding the protein, which are usually assigned to lipid molecules. The locations of the extra electron densities observed in this work did not significantly depend on the sample temperatures. Moreover, the positions of the extra electron densities on the present A_2A_R surface nearly overlapped with those of the electron densities corresponding to lipids in the MO-matrix based 4EIY model, suggesting that the lipid binding positions remain nearly unaltered in the two different matrix lipid environments.

Although the majority of the extra electron densities are rather obscure, we noted that five extra electron densities on the extracellular half of the A_2A_R surface can be assigned to two different lipid species, cholesterol and EROCOC_17+4_. The cholesterols are probably derived from the LCP crystallization matrix, whereas the possibility that it is derived from insect cell membrane cannot be ruled out. As shown in Fig. 3A, there are three cholesterol molecules, C1, C2, and C3, at the same locations as those of the three cholesterol molecules identified in the 4EIY model. Two EROCOC_17+4_ molecules, E1 and E2, are also depicted in Fig. 3A.

The electron density for E1, which is located near cholesterol C1, is well defined for the full EROCOC_17+4_ molecule: an isoprenoid chain with four methyl branches at the 5th, 9th, 13th, and 17th carbons and a hydrophilic erythritol connected via an ester bond (Fig. 3B and Sup. Figure 1A). Since IPCLs are not native lipids in the insect cells where the A_2A_R proteins were produced, the electron density for E1 can be unambiguously modeled as EROCOC_17+4_. Another EROCOC_17+4_ modeled with relatively clear electron densities for the methyl branches (E2 in Fig. 3A,C and Sup. Figure 1B) exists between helices I and VII. E2 may stabilize the N-terminal segment of helix I, as proposed for the corresponding lipid (OLA11) in the 4EIY model^16^. It is interesting that this lipid binding site corresponds to the antagonist/ligand binding site of the prostaglandin E receptor EP_4_, which may be on the ligand entry pathway^23^. In the 4EIY model, the lipids corresponding to E1 and E2 are MO and oleic acid, respectively. The electron densities for the three terminal carbons of the MO and the two terminal carbons of the oleic acid are weak, possibly due to the flexibility of the terminal regions of the oleic lipids.

### ***LCP-SFX data collection at 4 and 20*** °***C sample temperatures***

As described in the preceding section, the reliability factors of the data were improved by lowering the sample temperature. Table 1 also shows that the LCP-SFX data collection statistics depended on the sample temperature. For instance, the “crystal hit rate” (*Number of hit images/Number of collected images*) × 100 was as high as 46.0% for the LCP-SFX_4 °C, while it dropped to 20.2% for the LCP-SFX_20 °C, where the *hit image* is defined as an image with 20 or more diffraction spots, among all images collected at a 30 Hz frequency. The “% indexed images” (*Number of indexed images/Number of collected images*) × 100^4^ likewise displayed a similar trend, 32.8% and 18.5% for the LCP-SFX_4 °C and the LCP-SFX_20 °C, respectively. As the present LCP-SFX experiments were performed with the identical A_2A_R crystal-dispersed LCP, and except for the sample temperatures, were performed under the identical LCP-SFX experimental conditions (e.g., the injector nozzle diameter, the XFEL beam size, the pulse intensity, etc.), these results were most presumably ascribable to the different sample temperatures.

Table 2 lists the *D*_*4* °C_*/D*_*20* °C_ ratios estimated from the real-time LCP stream images, in the absence and presence of the XFEL beam, where *D*_*4* °C_ and *D*_*20* °C_ stand for the EROCOC_17+4_-LCP stream diameters at 4 and 20 °C at the XFEL beam intersection position, respectively. For reference, individual values of *D*_*4* °C_ and *D*_*20* °C_, are also listed. These values were estimated by assuming that the average LCP stream diameter at 20 °C in the absence of the XFEL beam was equal to the injector nozzle diameter *d*; i.e., *D*_*20* °C_ (beam off)_average_ ≡ 75 μm. It is first noted that the ratio in the absence of the beam, *D*_*4* °C_ (beam off)/*D*_*4* °C_ (beam off) ≈1.3. This suggests that the rheological properties of the EROCOC_17+4_-LCP are temperature dependent*.* In fact, we noted that the EROCOC_17+4_-LCP stream at 20 °C displayed “viscous fluid-like” behavior, whereas at 4 °C, “stiff or solid-like” features became conspicuous. This appears consistent with our empirical observation that the stress required to homogenize the EROCOC_17+4_-LCP progressively increases as the temperature is lowered. Moreover, the ratio in the presence of the beam, *D*_*4* °C_ (beam on)*/D*_*20* °C_ (beam on) ≈ 1.8, predicts that the “crystal hit rate” at 4 °C will be doubled as compared to that at 20 °C, in line with the observed “crystal hit rate”. This ratio also predicts the increased probability of multiple hits at 4 °C. The data in support of this prediction are actually seen in the “crystal index rate” (*Number of indexed images/Number of hit images*) × 100, with values of 71.3% at 4 °C and 91.5% at 20 °C.

**Table 2:** The LCP stream diameter ratio *D*_*4 °C*_*/D*_*20 °C*_ in the absence and presence of the XFEL beam.

XFEL beam	*d*/μm	*D*_*4 °C*_*/D*_*20 °C*_	*D*_*4 °C*_/μm^a^	*D*_*20 °C*_/μm^a^
Off	75	1.3	94 ± 3 (10)	75 ± 3 (12)
ON	75	1.8	135 ± 56 (65)	73 ± 20 (26)

*d*: Injector nozzle diameter, *D*_*4 °C*_*, D*_*20 °C*_: LCP stream diameters at 4 and 20 °C.

^a^Individual *D*_*4 °C*_, *D*_*20 °C*_ and standard deviation values, which were estimated by assuming that the average LCP stream diameter at 20 °C in the absence of the XFEL beam was equal to the injector nozzle diameter *d*; i.e., *D*_*20 °C*_ (beam off)_average_ ≡ 75 μm. The number of LCP stream diameter measurements performed every second is shown in parentheses.

Although the LCP stream was smooth and continuous in the absence of the XFEL beam, the beam-LCP stream intersection caused the LCP stream to exhibit quite dynamic and temperature-dependent behaviors in the presence of the XFEL beam. At 20 °C, the “viscous fluid-like” LCP stream displayed frequent fission and blowing-off of the stream at regular intervals, without significantly altering the *D*_20 °C_ (beam on) as compared to *D*_20 °C_ (beam off). On the other hand, at 4 °C the fission/blowing-off frequency of the “stiff or solid-like” LCP stream was reduced and occurred less regularly, tending to form an LCP lump with a larger *D*_4 °C_ (beam on) as compared to *D*_4 °C_ (beam off). Thus, the temperature-dependent LCP-SFX data collection statistics were most presumably ascribable to the modified response of the “viscous fluid-like” versus the “stiff or solid-like” LCP stream to the beam intersection.

To summarize, the LCP-SFX data collection at the 4 °C sample temperature could be performed with the higher “% indexed images” (32.8%) as compared to that at the 20 °C sample temperature (18.5%), and yielded more reliable diffraction data. The signal-to-noise ratio was also improved for the LCP-SFX_4 °C as compared to that for the LCP-SFX_20 °C; i.e., 8.3 and 7.6 at 4 and 20 °C, respectively, suggesting that the enhanced signal at 4 °C outweighed the presumed increase in the lipid-derived background diffraction from the LCP-stream with an increased D value. Therefore, the use of low temperature LCP samples could be a useful option for the LCP-SFX data collection.

### ***EROCOC***_***17***+***4***_*** and the EROCOC***_***17***+***4***_***-matrix are resistant to adopting the crystalline L***_C_*** phase***

Lipid/water systems generally exhibit a transition from a high temperature liquid crystalline to a low temperature crystalline L_C_ phase at a lipid-specific temperature, *T*_*K*_. Above *T*_*K*_, the lipid chains are in a fluid-like disordered conformation (a disordered chain state), with X-ray diffraction profiles characterized by a broad band around 4.6 Å, similar to a band observed from liquid paraffines^24^. Below *T*_*K*_, the lipid adopts a crystalline L_C_ phase, where the chains are fully extended and aligned parallel to each other, which is characterized by multiple sharp diffractions at all spacings from the small angle X-ray scattering (SAXS) to wide angle X-ray scattering (WAXS) regimes, reflecting the presence of long- and short-range three-dimensional orders in the molecular packing^24^.

**Figure 4:**
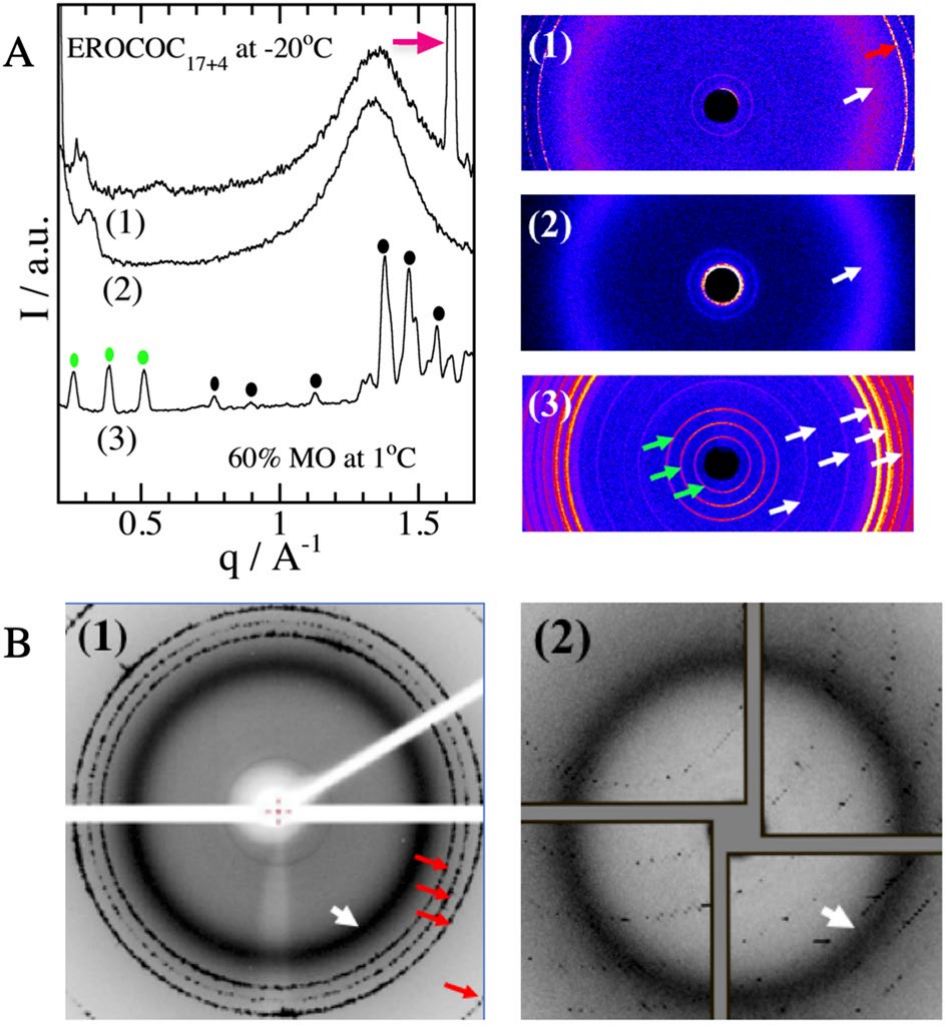
(**A**) X-ray diffraction profiles of 62.5% (w/w) EROCOC_17+4_/water (1) and dry EROCOC_17+4_ (2) at − 20 °C, together with the 2D-diffraction images used to construct each diffraction profile. The white and red arrows denote diffractions from disordered chains on the lipid and from ice, respectively. Profile (3) is from the MO-L_C_ phase [60% (w/w) MO/water] at 1 °C. The humps observed in profiles (1) and (2) below q ~ 0.35 Å^−1^ are due to x-ray leaks around the beam stopper. $q=\left(4\pi sin\theta \right)/\lambda$, where 2*θ* is the scattering angle and the wavelength λ = 1.54 Å. (**B**) (1) A diffraction image of the EROCOC_17+4_-matrix, 60% 9:1 (w/w) EROCOC_17+4_/cholesterol/water, at − 180 °C (measured under an evaporated liquid nitrogen flow). The broad band around 4.6 Å (white arrow) indicates that EROCOC_17+4_ remained in a disordered chain state at − 180 °C. The four red arrows denote hexagonal ice diffractions. (2) A diffraction image from the A_2A_R crystallization EROCOC_17+4_-matrix during the LCP-SFX_4°C data collection in a helium gas environment.

A typical example of an X-ray diffraction profile from an L_C_ phase is shown in Fig. 4A-(3), where the profile from the 60% (w/w) MO/water system at 1 °C is plotted. Note that the equilibrium MO/water system adopts the crystalline L_C_ phase below 17 °C^7^. Six representative peaks in the larger $q$ region are denoted by black dots in the profile and white arrows in the 2D image. The three sharp diffractions in the SAXS regime, denoted by the green dots in the profile and green arrows in the 2D-image in Fig. 4A-(3), are due to the 2nd, 3rd, and 4th diffractions of the MO-L_C_ phase with a lattice constant of 49.6 Å, which reasonably agrees with the reported value of 49.2 Å^25^.

Before discussing the phase behavior of the EROCOC_17+4_/water system at − 20 and around − 40 ~  to − 60 °C, let us briefly outline the phase behavior of the EROCOC_17+4_/water system above 0 °C^10^. The EROCOC_17+4_-LCP is identified in the lipid concentration ${W}_{Lipid}(=100-{W}_{Water})$ range from 60 to 80% (w/w) and over a temperature range from ca. 0 to ca. 55 °C [10, Sup. Table 1]. Thus, ${W}_{Lipid}$ = 60% (w/w) represents the water saturated EROCOC_17+4_-LCP composition. Once ${W}_{Lipid}$ is decreased below 60% (w/w), the excess water that can no longer be incorporated within the LCP separates out to adopt the “excess water + water saturated LCP” two-phase coexisting state. In the ${W}_{Lipid}$ range from 80 to ca. 92% (w/w), the system adopts a lamellar (L_α_) phase. In the highest ${W}_{Lipid}$ regime above ca. 93% (w/w), the phase structures have not yet been identified, due to the slow rate toward reaching the equilibrium state.

The X-ray diffraction profiles from the 62.5% (w/w) EROCOC_17+4_/water and dry EROCOC_17+4_ at − 20 °C are shown in Fig. 4A-(1) and (2), respectively. Both profiles are quite distinct from that of the MO-L_C_ phase in Fig. 4A-(3). They are characterized by a broad band around 4.7 Å, [$q(\equiv 2\pi /d)$ ~1.3 Å^−1^, white arrow in the 2D images], indicative of the disordered hydrocarbon chain of the lipid. The large peak at $q$= 1.62 Å^−1^ is the (100) diffraction of hexagonal ice (red arrows in the profile and the 2D image in Fig. 4A-(1))^26^, which disappeared above 0 °C (data not shown). No ice peak was observed in the dry EROCOC_17+4_ profile in Fig. 4A-(2), as expected. These results indicate that EROCOC_17+4_ remained in the disordered chain state (hence no L_C_ phase formation) at least down to − 20 °C, over a wide range of lipid hydration values (${W}_{Water}$) from fully hydrated 40% down to nearly 0% (w/w) water.

The adoption of the disordered chain state well below 0 °C is further supported by a differential scanning calorimetry study of the EROCOC_17+4_/water system^10^, which describes the effort in developing the L_C_ phase by cooling (ca. − 3 °C/min) the EROCOC_17+4_/water system from ca. 25 to − 60 °C, followed by a 3 h isothermal incubation at − 60 °C prior to the initiation of the heating run at a rate of 0.3 °C/min. As seen in the heating thermogram of 63.0% (w/w) EROCOC_17+4_ measured over a temperature range from − 40 to 15 °C (Sup. Figure 2A), only a single endothermic peak associated with melting of ice is present, indicating that EROCOC_17+4_ did not adopt the L_C_ phase at least down to − 40 °C^10^.

Let us next examine a possible phase state of the EROCOC_17+4_-matrix at a cryogenic temperature. To avoid excessively rapid cooling, the EROCOC_17+4_-matrix was first cooled slowly (ca. − 2 °C/min) from room temperature to − 20 °C, followed by 2–3 days of isothermal incubation prior to the incubation in liquid nitrogen (see X-ray scattering measurements section for details). We examined the diffraction profiles of twelve EROCOC_17+4_-matrix specimens at − 180 °C and confirmed that all of the specimens gave disordered chain profiles, as seen in Fig. 4B-(1), where a typical diffraction image of the EROCOC_17+4_-matrix (without A_2A_R) at − 180 °C, measured after a one-week incubation in liquid nitrogen, is shown. It displayed a broad band profile around 4.6 Å (white arrow), indicating that the EROCOC_17+4_-matrix did not adopt a crystalline L_C_ phase in liquid nitrogen, at least under the present experimental conditions. The four additional sharp diffractions at 3.87, 3.70, 3.44, and 2.66 Å (red arrows) are hexagonal ice diffractions^26^.

The possible phase structure of the EROCOC_17+4_/water system below 0 °C was inferred from the SAXS profiles of the 62.5% (w/w) EROCOC_17+4_ at − 20 °C and the 63% (w/w) EROCOC_17+4_ at − 60 °C. They both gave two peaks in a 2:1 ratio, 44.5 and 22.5 Å at − 20 °C and 46.8 and 23.4 Å at − 60 °C, respectively, suggesting the formation of an L_α_ phase with lattice constants of ${\xi }_{{L}_{\alpha }}\left(-20^\circ C\right)$ = 44.5 Å and ${\xi }_{{L}_{\alpha }}\left(-60^\circ C\right)$ = 46.8 Å, respectively. At − 180 °C, the four weak diffractions at 16.2, 12.2, 9.7, and 8.1 Å, observed in the SAXS region of Fig. 4B-(1), could also be compared with the 3rd, 4th, 5th and 6th diffractions of the L_α_ phase with the lattice constant of ${\xi }_{La}\left(-180^\circ C\right)$ = 48.0 Å. These results indicate that the EROCOC_17+4_/water and the EROCOC_17+4_-matrix/water systems most presumably adopted the L_α_ phase below 0 °C. For comparison, MO adopts the L_C_ phase at 1 °C (Fig. 4A-(3)), and eleven MO-matrixes subjected to the same cryo-cooling procedure displayed strong L_C_ diffractions at − 180 °C without exception, as shown in Sup. Figure 2.

It is therefore clear that EROCOC_17+4_ is far more resistant to the adaptation of the L_C_ phase as compared to MO. This allowed us to readily perform the LCP-SFX data collection at a 4 °C sample temperature in a helium gas atmosphere without any L_C_ phase formation. Figure 4B-(2) shows a typical in situ X-ray diffraction image of the EROCOC_17+4_-matrix captured during the LCP-SFX_4 °C data collection, in which the broad diffraction around 4.6 Å (white arrow) indicative of a fluid-like disordered chain state was seen, in addition to the diffraction spots from the A_2A_R crystals.

### ***EROCOC***_***17***+***4***_*** as a possible L***_***C***_*** formation-free crystal delivery medium***

In LCP-SFX, the viscous media injector streams the microcrystal-dispersed LCP inside the diffraction chamber, in either vacuum or helium gas conditions. In the case of a vacuum chamber at 20 °C, the MAG-matrix must be prepared from a short chain MAG, such as 9.7 MAG or 7.9 MAG, which are less prone to L_C_ phase formation than MO^3,4,6^; for instance, *T*_*K*_ of 7.9 MAG is 6 °C^27^. For crystals that only grow in the MO-LCP, it must be doped with one of the short chain MAGs just before loading into the injector to avoid the LCP → L_C_ phase transition upon evaporative cooling^3,4^.

Thus, the short chain 9.7 MAG and 7.9 MAG with the acyl chains of 16 carbon atoms-long serve as an L_C_ formation-free crystal delivery medium, whereas MO (9.9 MAG with the acyl chain of 18 carbon atoms-long, which yielded the most successful outcomes for a variety of MPs^2^, Fig. 1) does not. However, the measurements without extra matrix manipulation just before loading are more favorable. Moreover, it is well known that crystal growth and quality critically rely on the hydrocarbon chain length of the matrix lipid; e.g., according to Li et al., “a chain length disparity of just one carbon (in the 14- vs 15-carbon homologues) meant either getting or not getting crystals of outer membrane sugar transporter”^28^. Thus, the short chain MAGs may not be applicable to all MPs. The lipid with the acyl chain of 18 carbon atoms-long that serves as an L_C_ formation-free crystal delivery medium is useful.

It is often stated rather vaguely that ICPLs remain in the fluid state at low temperatures. However, as shown in Sup. Table 1, the *T*_*K*_ values (the lower temperature limit of the fluid state) and the temperature range over which the LCP is stable are lipid dependent. This is also true for the maximum hydration level of LCP (${W}_{Water}^{max}$) that determines the maximum protein buffer loading capacity of the LCP-matrix. Thus, IPCLs with these parameters favorable for the crystallization and crystal delivery medium should be explored.

EROCOC_17+4_ is akin to MO, in the sense that they both contain the acyl chain of 18 carbon atoms-long (Fig. 1), and the ${W}_{Water}^{max}$ value of 40% (w/w) (Sup. Table 1) is comparable to that of MO. Moreover, EROCOC_17+4_ forms the stable LCP over a temperature range from ca. 0 to ca. 55 °C [10, Sup. Table 1], and remains in the fluid state at least down to − 20 °C, over a wide range of hydration levels, ${W}_{Water}$ from 40% (w/w) to nearly 0% (w/w) dry state. Thus, EROCOC_17+4_ represents the first lipid with the acyl chain of 18 carbon atoms-long that meets the requirements for a matrix for MPs crystallization and an L_C_ formation-free crystal delivery medium without extra matrix manipulations.

## Concluding remarks

This study highlights the use of EROCOC_17+4_ as a new matrix lipid for A_2A_R crystallization and an A_2A_R crystal delivery medium in LCP-SFX. The results are summarized as follows.

1. The EROCOC_17+4_-matrix supports the growth of X-ray diffraction quality A_2A_R crystals and serves as a crystal delivery medium in LCP-SFX.
2. The optimum crystallization conditions for A_2A_R crystals in the EROCOC_17+4_-matrix did not differ significantly from those in the MO-matrix, and the EROCOC_17+4_-matrix could be manipulated by essentially the same experimental procedures as employed for the MAG-matrices. Thus, EROCOC_17+4_ can readily be accommodated within the current in meso crystallography experiments.
3. One noteworthy feature of EROCOC_17+4_ and the EROCOC_17+4_-matrix is the resistance to the adoption of the L_C_ phase. This makes it valuable for use in lower temperature crystallizations of MPs. In preliminary crystallization trials at 4 °C, we confirmed that the EROCOC_17+4_-matrix yielded A_2A_R crystals that diffracted to ca. 3 Å (not optimized), suggesting that EROCOC_17+4_ has potential for the in meso crystallization of thermally unstable MPs.
4. In LCP-SFX, EROCOC_17+4_ is the first lipid with the acyl chain of 18 carbon atoms-long that can serve as an L_C_ formation-free crystal delivery medium without extra matrix manipulations for low temperature data collection, and probably under vacuum chamber conditions as well. These features appear best suited for the lower temperature data collection of thermally unstable MP crystals, for which improved LCP-SFX diffraction data may also be expected.
5. As EROCOC_17+4_ is a member of the LCP-forming IPCL family^10–14^ (Sup. Table 1), IPCLs together with MAGs will expand the reliability/efficiency of in meso crystallization and serial microcrystallography. More details about the phase behavior and physical characteristics of EROCOC_17+4_ will be published elsewhere.

## Methods

### ***Expression and purification of A***_***2A***_***R***

A_2A_R harboring a bRIL, apocytochrome b562RIL from *Escherichia coli* (M7W, H102I, and R106L) fused in the third intracellular loop (ICL3), was prepared according to the method of Liu et al.^16^. Briefly, A_2A_R was expressed in Sf9 insect cells, using a baculovirus expression system. The A_2A_R was solubilized from the membrane fraction in 1% *n*-dodecyl-*β*-D-maltopyranoside (DDM, Anatrace) and 0.2% cholesteryl hemisuccinate (CHS, Sigma), with 4 mM theophylline (Sigma). The theophylline was substituted with 25 µM ZM241385 (Tocris and Sigma-Aldrich) on TALON cobalt-chelating resin (Clontech), in 20 mM HEPES buffer (pH 7.5) including 800 mM NaCl, 15 mM imidazole, 0.02% DDM, 0.004% CHS, and 10% glycerol, during affinity purification. The purified protein, in 20 mM HEPES buffer (pH 7.5) including 800 mM NaCl, 250 mM imidazole, 0.02% DDM, 0.004% CHS, 10% glycerol, and 25 µM ZM241385, was concentrated to 60 mg mL^-1^ (Amicon Ultra-4 100 kDa MWCO, Merck). The purity and monodispersity of the samples were assessed by analytical size exclusion chromatography (Sup. Figure 3).

### Crystallization

We performed ‘‘standard’’ in meso crystallization trials^2,29^ at 20 °C, with a 9:1 (w/w) mixture of EROCOC_17+4_ and cholesterol as the crystallization matrix. As described in Fig. 1, EROCOC_17+4_ is a mixture of 92% 1-*O*- and 8% 2-*O*-isomers. Typically, 9 mg of A_2A_R in buffer was homogenized with 13.5 mg EROCOC_17+4_-matrix [A_2A_R: EROCOC_17+4_-matrix = 40:60 ratio (w/w)] by a home-made mixing device, to obtain the viscous and transparent A_2A_R-LCP^30^.

A 40 nL portion of the homogenized A_2A_R-LCP was dispensed onto a siliconized glass plate with a 96-hole double stick sheet with a 135 µm thick spacer (Kajixx Co., Kawasaki, Japan)^30^, using a home-build robotic dispenser (Hato et al., to be published). The dispensed A_2A_R-LCP boluses were then covered with a glass coverslip after the addition of a series of 1 μL crystallization solutions. The 96-well glass plates were incubated at 20 °C in a RockImager 1000 (FORMULATRIX). The long axes of 10 crystals for the 30 and 32.5% PEG conditions (< 5 μm in size), 30 crystals for the 35% and 37.5% PEG conditions (~ 10 μm in size), and 50 crystals for the 40% PEG conditions (10 ~ 15 μm in size) were measured manually in bright field images (Sup. Figure 4), and the averages and standard deviations were determined. Crystal densities were also estimated by manually counting the separated and bright spots observed within a selected region of the whole (~ 40 nL) LCP bolus of cross polarized images: the whole area for the 40% PEG conditions, ~ 1/4 of the area for the 32.5–37.5% PEG conditions, and ~ 1/8 of the area for the 30% PEG conditions of corresponding LCP boluses (Sup. Figure 4). As the density values inevitably have large experimental uncertainties, they should be considered as rough estimates. The crystals for LCP-SFX were produced as described in the reference^31^, except that Ito MS-GAN025 (250 μL) microsyringes were used without mixing with any other lipid before the LCP-SFX measurement. As judged from the diffraction ability, the crystals were stable between 4–25 °C for at least a few weeks.

### Serial synchrotron rotation crystallography (SS-ROX)

SS-ROX data were collected at 100 K at SPring-8 BL32XU^32^. After breaking the cover glass with a glass cutter^29^, small crystals (~ 25 µm) in LCP were harvested with 200 µm cryo-loops (MiTeGen) and flash-cooled without additional cryo-protectant. Using a 5 × 5 µm^2^ beam, an entire cryo-loop was scanned with 0.5° rotation and 0.45 s per image with a photon flux of 6.7 × 10^11^ photons/s at a wavelength of 1.0000 Å, using an EIGER X 9 M detector (DECTRIS)^20^. The experiment was performed automatically by the ZOO system^33^. Fourteen cryo-loops provided 6346 hits with more than 20 spots in an image, identified using the Cheetah program^34^ adapted for EIGER, and 3701 hits were indexed using kamo.single_images_integration^20^ with XDS^35^. Among the indexed images, 2100 images were selected based on the highest average *I*/σ(*I*). Intensities were merged using kamo.merge_single_images_integrated^20^ in a Monte-Carlo fashion, as in CrystFEL^36^, by discarding low-partiality reflections.

### Lipidic cubic phase serial femtosecond crystallography (LCP-SFX)

LCP-SFX data were collected using the 20 and 4 °C samples at SACLA^37^ BL3^38^ EH4c (LCP-SFX_4 °C and _20 °C, Table 1). For each measurement, a ~ 25 µL A_2A_R-LCP sample was transferred into a dedicated sample injector with a 75 µm ϕ nozzle. The sample temperature was controlled by a temperature variable liquid circulator (OHM Electric Co., Ltd.) connected to the sample injector. For the data collection using the 4 °C sample, the sample injector and the circulating hose were covered with urethane and rubber jackets, respectively, to maintain the temperature and prevent condensation. The sample was injected at a rate of 0.24 µL min^−1^ into a 30 Hz X-ray laser pulse, to achieve a 30 µm inter-pulse distance at the injected sample. To achieve a smooth flow, a room temperature helium sheath gas (0.6 L min^−1^) was applied around the injected sample, and aspirated (0.8 L min^−1^) just below the sample. The X-ray energy was 10.1 keV (1.23 Å wavelength), the beam intensity was ~ 560 µJ per pulse, and the data were collected with a multiport CCD^39^, Phase III, with improved quantum efficiency at a higher energy X-ray region around 12 keV with a thicker (300 µm) sensor. Less than 100 min were needed to obtain a complete data set with the 25 µL A_2A_R-LCP samples (31,312 and 46,877 indexed images for 20 and 4 °C, respectively). Data collection was guided by the data processing pipeline for SACLA^40^, based on Cheetah^34^ and CrystFEL 0.6.2^36^. Images with 20 or more spots detected by Cheetah were considered as hits and processed by CrystFEL. DirAx 1.16^41^ was used for indexing. Integrated intensities were merged by process_hkl in the CrystFEL suite, with linear scaling and the per-image resolution cutoff (the “–push-res 1.5” option).

### Structural refinement

The crystal structure of MO-matrix-grown A_2A_R (PDB ID: 4EIY) was employed as the starting model. Models were rebuilt by iterative manual modifications in COOT^42^ and refinement by REFMAC5^43^ in CCP4^44^. Structural figures were prepared with PyMOL (Schrödinger, L. L. C. The PyMOL Molecular Graphics System. Version 1.8). The stereochemistry of the protein structures was checked by Rampage^45^, and no amino acid residues were in the disallowed region of the Ramachandran plot. Cholesterol and EROCOC_17+4_ models were introduced where the shape and size of the electron density fit well. For the EROCOC_17+4_ stereochemistry, (2R,3S) was applied to the erythritol moiety, and the 5,9,13,17-tetramethyloctadecanoyl moiety was modeled as the All R (5R,9R,13R) conformation, as in β-XylOC_16+4_^15^. Linear hydrocarbons were modeled for elongated electron densities that could not be characterized well as a specific lipid. Polder maps^46^, which exclude the bulk solvent around the individually omitted lipids E1, E2, C1, C2 and C3, were prepared with Phenix^47^.

### X-ray scattering measurements

The X-ray scattering measurements of dry EROCOC_17+4_, 62.5% (w/w) EROCOC_17+4_/water at − 20 °C, and 60% (w/w) MO/water at 1 °C were performed with a Rigaku Nano-Viewer, using Ni-filtered CuKα radiation (λ = 1.54 Å) generated by a Rigaku FR-E unit (45 kV, 45 mA) with a triple pinhole collimator (0.4 mmϕ × 0.2 mmϕ × 0.45 mmϕ). Sample-to-detector distances of 92 mm (WAXS) or 448 mm (SAXS), were calibrated by using lead stearate^48^ and stearic acid^49^, respectively. The sample temperature was controlled with an UT4040-PF Peltier module (Ampère, Tokyo) at − 20 ± 0.5 °C and 1 ± 0.1 °C, monitored by a Pt-100 thermoresistor (Hayashi Denko, Pt-100-A-1-M, Tokyo). The two-dimensional powder diffractions were recorded by a PILATUS100K-S detector (DECTRIS, Switzerland) and analyzed by a Rigaku 2-DP data processing system. The data collection of 63.0% (w/w) EROCOC_17+4_/water at −60 °C was performed with Ni-filtered CuKα radiation (λ = 1.54 Å) from a Rigaku RU-200 X-ray generator (40 kV, 100 mA) with a double pinhole collimator (0.5 mmϕ × 0.3 mmϕ), and the sample temperature was controlled with a Mettler FP82HT temperature control-stage^10,11^.

The samples (except for dry lipid) were homogenized by using a homebuilt mixing device consisting of a pair of MS-GAN025 (250 μl) microsyringes (Ito Co., Fuji, Japan) with a monolithic stainless-steel coupler^10,30^. The homogenized sample was then transferred into quartz capillaries (1.5 mm in diameter, Glass Berlin) and immediately flame sealed and glued with 5-min epoxy (Konishi Co., Ltd. Osaka). To fully develop a sub-zero degree temperature phase at − 20 °C (the L_α_ phase), the EROCOC_17+4_ samples were first cooled (ca. − 2 °C/min) from room temperature to − 20 °C, followed by one day of isothermal incubation at − 20 °C (Medicool, Sanyo Electric Co., Ltd., Osaka) prior to the − 20 °C data collection. The − 20 °C isothermal incubation for up to three weeks did not alter the results. The MO sample was first incubated at − 20 °C to fully develop the L_C_ phase, and then the sample temperature was increased to 1 °C prior to the data collection at 1 °C.

The X-ray scattering profiles at − 180 °C of the EROCOC_17+4_- and MO-matrixes, 60% 9:1 (w/w) EROCOC_17+4_/cholesterol/water and 60% 9:1 (w/w) MO/cholesterol/water, were measured by a Rigaku FR-X unit (45 kV, 66 mA, CuKα radiation λ = 1.54 Å) with a sample to PILATUS200K detector distance of 70 mm, calibrated by using lysozyme crystals. The image data were analyzed by the Rigaku Crystal Clear data processing system.

The EROCOC_17+4_- and MO-matrixes in a 200 µm cryo-loop (MiTeGen) were placed in a hermetically sealed plastic container, which contained a small amount of water at the bottom to ensure that the matrix specimens were maintained in the water saturated phase condition. To avoid excessively rapid cooling, the matrix specimens were first cooled (ca. − 2 °C/min) from room temperature to − 20 °C, followed by 2–3 days of − 20 °C isothermal incubation to fully develop a sub-zero degree temperature phase (the L_α_ phase), and were then incubated in liquid nitrogen. The − 180 °C measurements were performed under an evaporated liquid nitrogen flow, after 1- and 7-day incubation durations in liquid nitrogen. Both diffraction profiles were indistinguishable.

## Supplementary information

Supplementary Information

## Data Availability

Raw diffraction images of LCP-SFX have been deposited in the CXIDB, as entry 140. Raw diffraction images of SS-ROX_cryo are available at the Zenodo data repository (10.5281/zenodo.3595784). The A_2A_R crystal structure models of LCP-SFX_4 °C, LCP-SFX_20 °C, and SS-ROX_cryo were deposited in the Protein Data Bank, under the accession codes 6LPJ, 6LPK, and 6LPL, respectively.
