# Supplementary material for: Isoprenoid-chained lipid EROCOC17+4: a new matrix for membrane protein crystallization and a crystal delivery medium in serial femtosecond crystallography
Source: Sci Rep. 2020 Nov 9;10:19305. doi: 10.1038/s41598-020-76277-x (PMC7652841; doi:10.1038/s41598-020-76277-x)
Supplement: Supplementary file 1 — Supplementary Information [file 41598_2020_76277_MOESM1_ESM.pdf]

Supplementary information for

Isoprenoid-chained lipid EROCOC$_{17+4}$ – a new matrix for membrane protein crystallization and a crystal delivery medium in serial femtosecond crystallography

Kentaro Ihara, Masakatsu Hato, Takanori Nakane, Keitaro Yamashita, Tomomi Kimura-Someya, Toshiaki Hosaka, Yoshiko Ishizuka-Katsura, Rie Tanaka, Tomoyuki Tanaka, Michihiro Sugahara, Kunio Hirata, Masaki Yamamoto, Osamu Nureki, Kensuke Tono, Eriko Nango, So Iwata, and Mikako Shirouzu

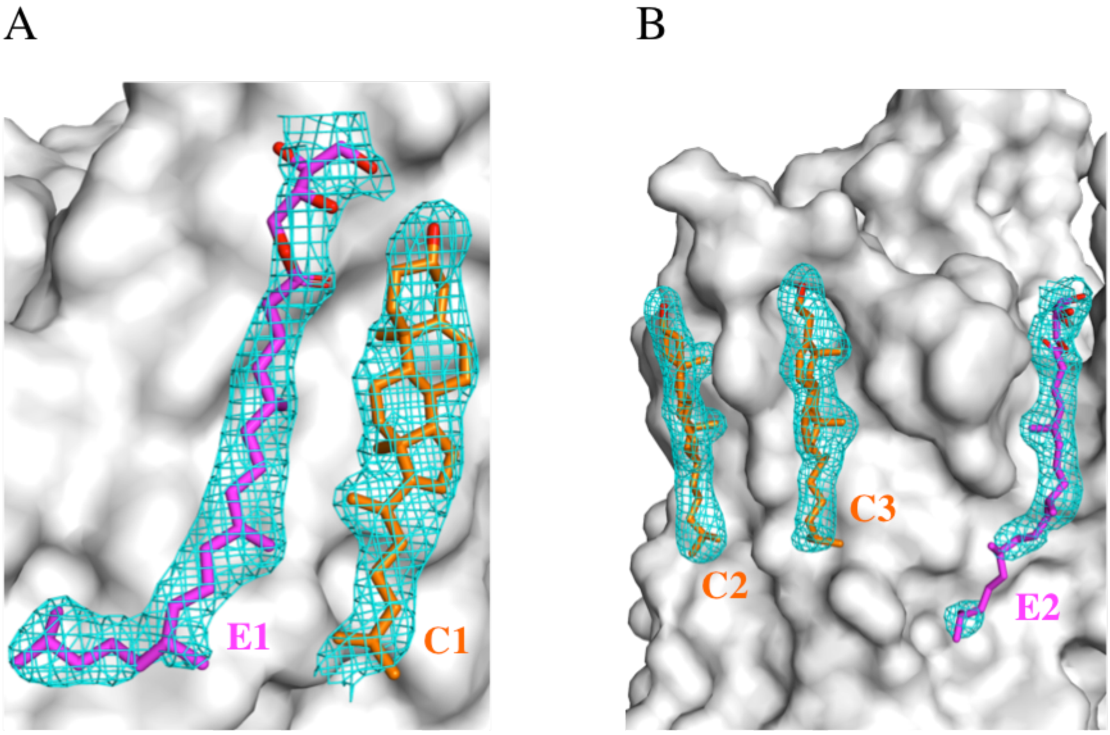

Supplementary Figure 1. The Polder-omit map around lipids E1 and C1 (A), and E2, C2 and C3 (B) on the molecular surface of LCP-SFX_4°C. The areas drawn in panels A and B are the same as panels B and C of Figure 3, respectively. The mF_o-DF_c electron density maps around the lipid models are contoured at 3.0 σ (cyan mesh). This figure is prepared by PyMOL Ver. 1.8 (https://pymol.org).

A

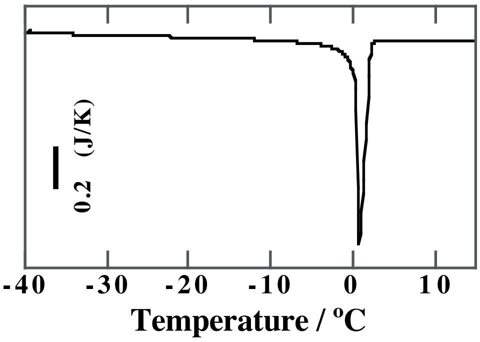

B

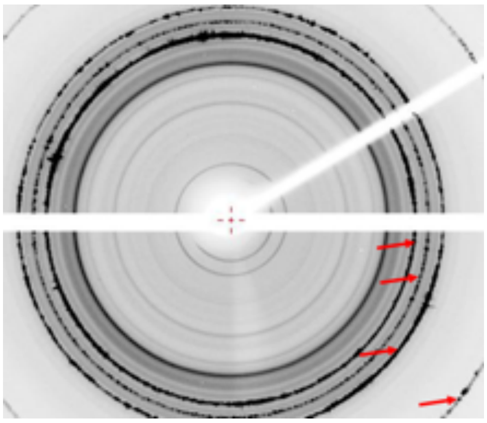

Supplementary Figure 2. (A) The heating DSC thermogram of the 63.0 % (w/w) EROCOC$_{17+4}$ measured over a temperature range from -40 to 15°C. The samples were incubated at -60°C for 3h before initiating the heating scan at a rate of 0.3°C/min. The large endothermic peak around 0°C is due to the melting of ice (see text for more details). (B) A typical diffraction image of the MO-matrix, 60 wt% 9:1 (w/w) MO/cholesterol/water, at -180°C (measured under an evaporated liquid nitrogen flow), which is characterized by multiple sharp diffractions at all spacings, reflecting the presence of long- and short-range three-dimensional orders of the MO-L$_C$ phase. The four red arrows denote hexagonal ice diffractions.

A

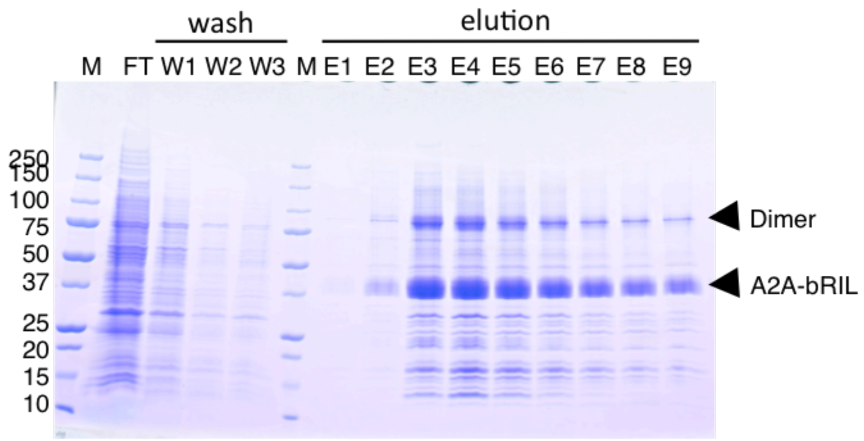

B

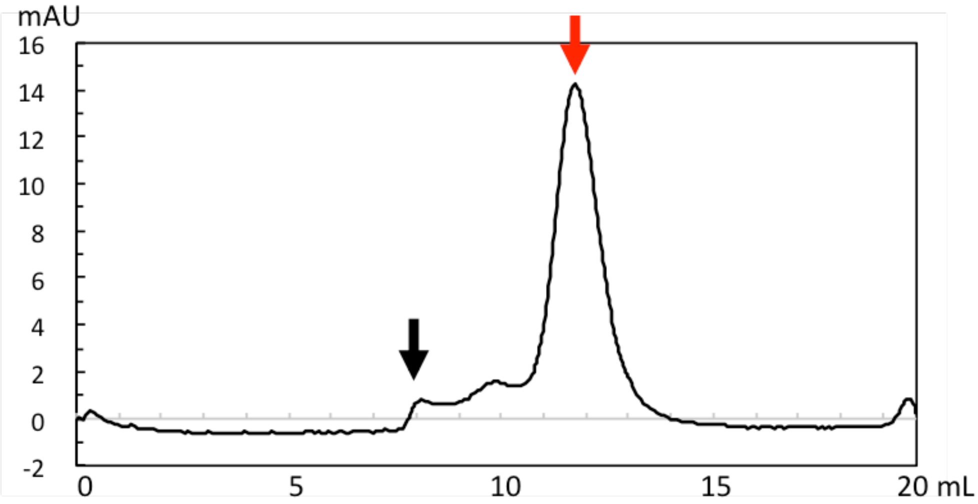

Supplementary Figure 3. (A) SDS-PAGE analysis of cobalt-affinity chromatography fractions. Arrowheads indicate the dimer and monomer of A_{2A}R-bRIL fusion proteins. E3-E5 elution fractions were collected and concentrated. (B) Analytical size-exclusion chromatography (aSEC) of the sample used for crystallization. Black and red arrows indicate the void volume and the peak of the A_{2A}R proteins, respectively. The aSEC using Superdex 200 (GE Healthcare) confirmed that the sample is almost completely monodisperse, and the apparent molecular weight is about 160 kDa as calculated from the retention volume.

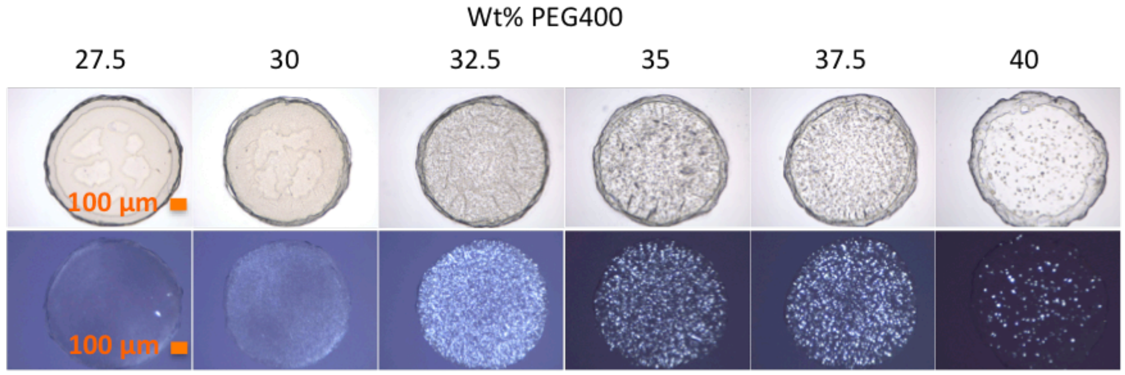

Supplementary Figure 4. Entire well images of bright field (upper) and cross polarized (lower) crystal visualizations in a 96-well glass screening plate. The size and density of the crystals were calculated using the images.

Supplementary Table 1. Temperature-dependent phase behavior, crystalline L_C → liquid crystalline (LC_I) phase transition temperature (T_K), liquid crystalline (LC_I) → liquid crystalline (LC_{II}) phase transition temperature (T_{tr}), and maximum hydration amount of the LCP (W_{Water}^{max}) at 25°C.

Lipid	Phase sequence[a]	T_K/°C	T_{tr}/°C	W_{Water}^{max} / % (w/w)	Ref.
Ester-type IPCLs					
$GlyOCOC_{13+3}$ 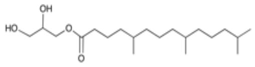	LCP → H_{II}	< 0[b]	28 ± 3	25 ± 3	[1,2]
$EROCOC_{17+4}$ 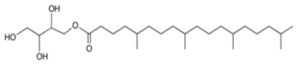	LCP → H_{II}	< 0[b]	55 ± 3	40 ± 2	[1,2]
$EROCOC_{15+4}$ 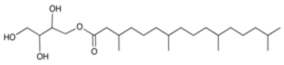	LCP	< 0[b]	> 65	40 ± 3	[1,2]
$PEOCOC_{17+4}$ 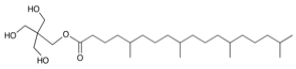	L_α → LCP	-17	35 ± 3	42 ± 2[c]	[1,2]
$PEOCOC_{15+4}$ 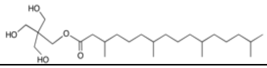	L_α → LCP	-5.5	10 ± 10	~ 40	[1,2]
Ether-type IPCLs					
$EROC_{14+3}$ 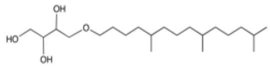	L_α → LCP	n.m.	20 ± 5	53 ± 3	[1,2]
$EROC_{12+3}$ 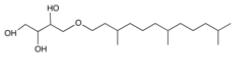	L_α → LCP	< 0[b]	15 ± 5	n.m.	[1,2]
$PEOC_{18+4}$ 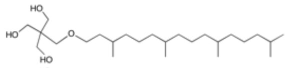	LCP → H_{II}	-33	33 ± 3	25 ± 3	[1,2]
$PEOC_{16+4}$ 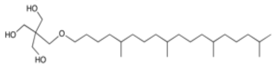	LCP → H_{II}	-34	40 ± 5	25 ± 3	[1,2]
$PEOC_{14+3}$ 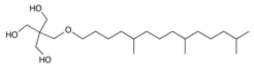	L_α → LCP	< 0[b]	33 ± 3	n.m.	[1,2]
$PEOC_{12+3}$ 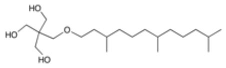	L_α → LCP	n.m.	38 ± 3	n.m.	[1,2]
β-$XylOC_{18+4}$ 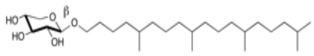	LCP → H_{II}	-13	50 ± 5	35 ± 2	[1,2]
β-$XylOC_{16+4}$ 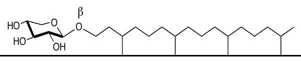	L_C → LCP	9	76 ± 2	35 ± 1	[3]
Amide-type IPCLs					
$GlyNCOC_{15+4}$ 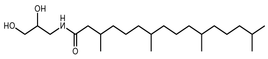	LCP	-	-	-	[4]
$GlyNMeCOC_{15+4}$ 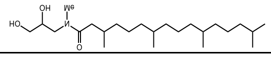	LCP	-	-	-	[4]

[a]: Phase sequence of the most diluted liquid crystalline phase as the temperature was increased from 0 to 65°C. L_α: lamellar phase, LCP: inverted cubic phase, H_{II}: inverted hexagonal phase, L_C: crystalline phase.

[b]: 3 h incubation of the sample at –60°C failed to induce the L_C phase. n.m.: not measured. W_{Water}^{max}: value at 25°C, which was estimated from the lattice constant vs. the lipid concentration curve.
[c]: Value of a super cooled LCP at 25°C.

The isoprenoid chain is abbreviated as Cp+q, where p and q stand for the number of carbon atoms in the longest unbranched carbon chain in the molecule (excluding the carbonyl carbon) and the number of methyl branches along it, respectively. The headgroups are abbreviated as glycerol (Gly), erythritol (ER), pentaerythritol (PE), and xylose (Xyl). For instance, EROCOC$_{17+4}$ represents a lipid molecule, in which the C$_{17+4}$ chain is linked to the erythritol head group (ER) via an ester linkage (OCO).

References

(1) Yamashita, J., Shiono, M. & Hato, M. New lipid family that forms inverted cubic phases in equilibrium with excess water: molecular structure−aqueous phase structure relationship for lipids with 5,9,13,17-tetramethyloctadecyl and 5,9,13,17-tetramethyloctadecanoyl chains. *J. Phys. Chem. B* **112,** 12286–12296 (2008).

(2) Hato, M., Yamashita, J. & Shiono, M. Aqueous phase behavior of lipids with isoprenoid type hydrophobic chains. *J. Phys. Chem. B* **113,** 10196–10209 (2009).

(3) Hato, M., Yamashita, J., Kato, T. & Abe, Y. Aqueous phase behavior of a 1-*O*-phytanyl-β-D-xyloside/water system. Glycolipid-based bicontinuous cubic phases of crystallographic space groups *Pn*3*m* and *Ia*3*d*. *Langmuir* **20,** 11366–11373 (2004).

(4) Ishchenko, A., *et al.* Chemically stable lipids for membrane protein crystallization. *Cryst. Growth Des*. **17,** 3502–3511 (2017).
